# Dysphagia risk, low muscle strength and poor cognition predict malnutrition risk in older adults athospital admission

**DOI:** 10.1186/s12877-018-0771-x

**Published:** 2018-03-21

**Authors:** Idah Chatindiara, Jacqueline Allen, Amy Popman, Darshan Patel, Marilize Richter, Marlena Kruger, Carol Wham

**Affiliations:** 1 School of Food and Nutrition, Massey University, Auckland, New Zealand; 2 Department of Surgery, University of Auckland, Auckland, New Zealand

**Keywords:** Older adults, Hospital malnutrition risk, Dysphagia risk, Muscle strength, MNA®-SF

## Abstract

**Background:**

Malnutrition in patients admitted to hospital may have detrimental effects on recovery and healing. Malnutrition is preceded by a state of malnutrition risk, yet malnutrition risk is often not detected during admission. The aim of the current study was to investigate the magnitude and potential predictors of malnutrition risk in older adults, at hospital admission.

**Methods:**

A cross-sectional was study conducted in 234 older adults (age ≥ 65 or ≥ 55 for Māori or Pacific ethnicity) at admission to hospital in Auckland, New Zealand. Assessment of malnutrition risk status was performed using the Mini Nutritional Assessment Short-Form (MNA®-SF), dysphagia risk by the Eating Assessment Tool (EAT-10), muscle strength by hand grip strength and cognitive status by the Montreal Cognitive Assessment (MoCA) tool.

**Results:**

Among 234 participants, mean age 83.6 ± 7.6 years, 46.6% were identified as at malnutrition risk and 26.9% malnourished. After adjusting for age, gender and ethnicity, the study identified [prevalence ratio (95% confidence interval)] high dysphagia risk [EAT-10 score: 0.98 (0.97–0.99)], low body mass index [kg/m^2^: 1.02 (1.02–1.03)], low muscle strength [hand grip strength, kg: 1.01 (1.00–1.02)] and decline in cognition [MoCA score: 1.01 (1.00–1.02)] as significant predictors of malnutrition risk in older adults at hospital admission.

**Conclusion:**

Among older adults recently admitted to the hospital, almost three-quarters were malnourished or at malnutrition risk. As the majority (88%) of participants were admitted from the community, this illustrates the need for routine nutrition screening both at hospital admission and in community-dwelling older adults. Factors such as dysphagia, unintentional weight loss, decline in muscle strength, and poor cognition may indicate increased risk of malnutrition.

## Background

The current life expectancy at birth (81.7 years) for New Zealand is among the top life expectancies within the Organisation for Economic Co-operation and Development countries [1]. This raises the issue of whether efficient strategies have been put in place to ensure this growth in life expectancy will go along with good quality of life [2, 3]. Since ageing is a well-documented risk factor for hospitalisation [4–6], there is great need to come up with strategies that help older people to continue living healthily in the community. Although ageing is inexorable, several factors contributing to hospital admission in older adults can be modified, for example, nutrition status. Despite being well documented as a preventable condition, hospital malnutrition is highly prevalent among older adults (20–60%) [7–9]. It remains unclear whether malnutrition occurs before or after admission since at hospital admission, nutrition screening is not mandatory and is conducted infrequently [10–12].

Malnutrition is preceded by a state of malnutrition risk thus nutrition risk screening may identify those who show nutritional compromise before they reach a state of malnutrition, enabling intervention and prevention. Although several international studies assessed malnutrition risk at hospital admission [13–21], the wide range of malnutrition risk prevalence (15–78%) observed from these studies raises questions. Differences in methodology used to assess malnutrition risk may partly explain the reported discrepancies. Several methods of malnutrition risk screening have been established and the choice of method used should consider participants age and setting [22, 23]. The Mini Nutritional Assessment Short-Form, (MNA®-SF) is a validated tool for assessment of malnutrition risk in older adults in multiple settings, including the hospital [24]. The MNA®-SF is used globally and has a sensitivity of 89% and specificity of 82% for assessing malnutrition risk in older adults [22, 25].

Studies suggest that malnutrition risk factors are multidimensional. These include sociodemographic (e.g. age, gender, living arrangements and employment status), psychological (e.g. individuals’ thoughts and feelings), and health status (e.g. functional ability, presence of comorbidities, polypharmacy, impaired chewing and swallowing) [26–30]. Understanding these factors may be helpful for identifying those at malnutrition risk and providing timely preventative interventions. Levels of cognitive impairment often increase with ageing [31], and have the potential to influence nutrition status. Previous studies have reported positive associations between satisfactory dietary intake and better cognition [32]. Furthermore, older adults are more vulnerable to dysphagia (swallowing difficulties) [33], which can lead to malnutrition through decline in food intake. Among healthy community-dwelling older adults, a positive correlation between posterior tongue strength (swallowing strength) and hand grip strength (a measure of muscle strength) was observed [34]. This suggests that loss of muscle strength at one site e.g. hand grip, may be indicative of weakness at other sites e.g. oral cavity. To date, there is limited literature supporting a strong association between tongue strengthening and swallow outcomes. However, among older adults in long term care, reduced tongue strength was found to be associated with observable signs of swallowing difficulty and reduced food consumption [35]. Therefore, exploring associations between muscle strength, dysphagia and malnutrition appears warranted.

In New Zealand, malnutrition risk screening is not mandatory and knowledge of prevalence of malnutrition in older adults is lacking. Using the MNA®-SF, the aim of the current study was to investigate the magnitude and potential predictors of malnutrition risk in older adults, at hospital admission.

## Methods

### Setting and sample

A cross-sectional study was conducted in two hospitals in Auckland, New Zealand. The study included a non-randomised convenience sample of 234 older adults. Recruitment was at hospital admission between July 2014 and September 2015. Ethics approval was granted by the Health and Disability Ethics Committee: Northern A region 14/NTA/70/AMO1.

At hospital admission, participants were provided with an information sheet, the study procedures were explained and written consent was obtained. The inclusion criteria were: age ≥ 65 or ≥ 55 for Māori or Pacific ethnicity, admission to hospital rehabilitation wards within the preceding 5 days, able to comprehend study requirements and give consent. A younger age group was included for Māori and Pacific participants as they have a lower life expectancy compared to other ethnicities [36]. Furthermore, findings on health disparities related to ethnicity in New Zealand indicates that Māori and Pacific populations are more likely to biologically age earlier due to poor health [37, 38]. Participants were excluded if they presented with dysphagia, known gastrointestinal disease, known pre-existing cognitive impairment, malignancy or tracheostomy.

### Assessments

Sociodemographic data including age, gender, ethnicity, marital status, living arrangements, pension-related income and highest education level attained were obtained from participants and clinical notes. Participants were asked if they required help with activities of daily living (ADL) to understand if individuals may be limited in physical functioning. Comorbidities, use of medications and nutritional supplements were evaluated from participant reports and clinical notes. Self-reported dental status was recorded as either dentate (able to chew food without appliances), edentulous (missing teeth contributing to chewing problems and not using dental appliance) or use of dental appliances. Validated tools for screening malnutrition (MNA®-SF), dysphagia risk (Eating Assessment Tool-10 (EAT-10)) and cognitive status (Montreal Cognitive Assessment (MoCA)) in older adults were used. Furthermore, physical measurements specifically weight, height, body mass index (BMI) and muscle strength were done.

### Malnutrition risk status

The MNA®-SF is comprised of six items that assess food intake, weight loss, mobility, psychological stress, neuropsychological problems, and BMI. Using the previously validated MNA®-SF cut-off points; nutrition status was defined as the state of being either well-nourished (MNA®-SF score ≥ 12), at malnutrition risk (MNA®-SF score 8–11) or malnourished (MNA®-SF score 0–7). Furthermore, malnutrition risk status was categorised as: ‘at risk’ (MNA®-SF score ≤11) and ‘not at risk’ (MNA®-SF score ≥ 12). Lower MNA®-SF score indicate increased malnutrition risk. All assessments were performed per the MNA®-SF user guide [24].

### Dysphagia risk

EAT-10, a self-reported validated questionnaire that assesses perception of swallowing difficulty was used to evaluate dysphagia risk. Increase in the EAT-10 score is indicative of increased dysphagia risk or swallowing difficulties, and an EAT-10 score ≥ 3 is suggestive of dysphagia [39].

### Cognitive status

Cognitive status was determined using the MoCA tool. This tool is a multi-domain test which enables detection of mild cognitive impairment through several assessments including memory, attention, conceptual thinking and orientation. Low MoCA scores indicate decline in cognitive status and a score ≤ 26 indicates cognitive impairment [40].

### Muscle strength

Muscle strength was assessed using hand grip strength [41]. A hand dynamometer (Jamar Hydraulic Dynamometer, model #5030 J1, Sammons Preston, USA) was used. The measurement procedure used conformed to the standard approved by the American Society of Hand Therapists [42]. The mean of six measurements, three from each hand was recorded. In participants (*n* = 8) who could only provide measurements from one hand, a mean of three measurements was used from the dominant hand [42, 43].

### Statistical analysis

The main outcome measure was malnutrition risk indicator score (MNA®-SF). Continuous data was assessed for normality using Shapiro Wilcoxon tests and through visual analysis of the normality plots. Descriptive statistics for normally distributed data were presented as mean ± standard deviation (SD) and categorical data were presented as frequencies and percentages. Chi-square test of independence or Fisher exact tests was used to analyse the association between participants’ demographic characteristics and malnutrition status. As the prevalence of malnutrition risk was higher than 10%, Poisson regression with robust variance estimation was used to quantify the magnitude of the associations between potential predictors and malnutrition risk (MNA®-SF score) [44]. Each characteristic was added to the Poisson regression model adjusting for the major non-modifiable human factors (age, gender and ethnicity), medications and comorbidities, as these factors are related to malnutrition risk. The magnitudes of associations were reported as prevalence ratio (PR) and 95% confidence interval (95% CI). Results were considered significant at *p* < 0.05. All statistical analyses were done using IBM SPSS Statistics version 24.

## Results

### Study population and prevalence of malnutrition risk

Malnutrition risk assessment was completed in 234 participants aged (mean years ± SD) 83.6 ± 7.6, of whom 40.6% were women. Eighty-eight percent of participants were admitted from the community and 41% had a BMI less than 23 kg/m^2^, which is below the proposed healthy cut-off point (BMI ≥23kg/m^2^) for older adults [45]. Data collection was conducted within 5 days of hospital admission, regardless of admission reason. No adverse events were encountered during the study data collection. Table 1 shows the participants characteristics by nutrition status.

**Table 1:** Participants’ demographic characteristics by malnutrition status: MNA®-SF

Characteristics		Malnutrition status		
	*Total	Well-nourished	At malnutrition risk	Malnourished
	*n* (%)	*n* (%)	*n* (%)	*n* (%)
All participants’	234 (100)	62 (26.5)	109 (46.6)	63 (26.9)
Admission source				
- Community	156 (88.1)	46 (29.5)	70 (44.9)	40 (25.6)
- Aged care^a^	21 (11.9)	5 (23.8)	7 (33.3)	9 (42.9)
Age				
- < 85 years	122 (52.1)	32 (26.2)	58 (47.5)	32 (26.2)
- ≥85 years	112 (47.9)	30 (26.8)	51 (45.5)	31 (27.7)
BMI				
- < 23 kg/m^2^	95 (40.6)**	9 (9.5)	45 (47.4)	41 (43.2)
- ≥23 kg/m^2^	139 (59.4)	53 (38.1)	64 (46.0)	22 (15.8)
Gender				
- Men	95 (40.6)	25 (26.3)	46 (48.4)	24 (25.3)
- Women	139 (59.4)	37 (26.6)	63 (45.3)	39 (28.1)
Marital status				
- Married/partnered	95 (40.6)	26 (27.4)	42 (44.2)	27 (28.4)
- Single^b^	139 (59.4)	36 (25.9)	67 (48.2)	36 (25.9)
Ethnicity				
- All other ethnicities^c^	87 (37.2)	21 (24.1)	38 (43.7)	28 (32.2)
- New Zealand European	147 (62.8)	41 (27.9)	71 (48.3)	35 (23.8)
Living arrangements				
- Living alone	104 (44.4)	31 (29.8)	49 (47.1)	24 (23.1)
- Living with partner only	73 (31.2)	21 (28.8)	32 (43.8)	20 (27.4)
- Living with others	57 (24.4)	10 (17.5)	28 (49.1)	19 (33.3)
Income source				
- Pension only income	152 (65.0)	37 (24.3)	69 (45.4)	46 (30.3)
- Pension plus other income	82 (35.0)	25 (30.5)	40 (48.8)	17 (20.7)
Education				
- Primary	52 (22.2)	11 (21.2)	28 (53.8)	13 (25.0)
- Secondary	144 (61.5)	38 (26.4)	62 (43.1)	44 (30.6)
- Tertiary	38 (16.2)	13 (34.2)	19 (50.0)	6 (15.8)

*BMI* body mass index, *MNA®-SF* Mini Nutritional Assessment Short-Form

*The percentages (%) in the total column are percentage total. All other percentages are percentage total within a category. All values are reported after rounding up to 1 decimal place, therefore percentages add up to 100 ± 0.1 **Only BMI was statistically significant across nutrition status groups: *p* < 0.05 Chi-square test of independence

^a^Aged care: admitted from long term residential care (*n* = 16) or private hospital interim care (*n* = 5)

^b^Marital status single: widowed *n* = 110, divorced *n* = 15, single/never married *n* = 14

^c^Other ethnicities: Māori *n* = 6, Pacific n = 6, others *n* = 75

The study identified 109 participants (46.6%) at malnutrition risk (MNA®-SF score 8–11) and 63 participants (26.9%) as malnourished (MNA®-SF score 12–14).

### Physical and health predictors of malnutrition risk

Table 2 summarises the descriptive data for predictive factors evaluated in relation to malnutrition risk, and the respective magnitudes [PR (95% CI)] of association with malnutrition observed. From the descriptive data, participants showed a general decline in physical and health status. Only a third maintained full dentition and an increased risk of swallowing problems was suggested by the elevated mean EAT-10 score of 3.0 (± 5.8). A majority of the participants had reduced muscle strength (hand grip), reduced cognitive functioning scores, required daily help with ADL and had approximately six comorbidities. Hypertension (and other vascular diseases), osteoporosis and chronic obstructive pulmonary disease were the most frequently observed conditions.

**Table 2:** Physical and health predictors of malnutrition risk indicator score (MNA®-SF)

	Descriptive statistics^a^			Malnutrition risk score^b^	
	(*n* = 234)			(MNA®-SF)	
	Total	Not at risk (*n* = 62)	At risk (*n* = 109)	Prevalence ratio (95% CI)	*p* value
Dental status n (%)					
Dentate	46 (19.7)	13 (28.3)	33 (71.7)	1	
Edentulous	51 (21.8)	13 (25.5)	38 (74.5)	0.95 (0.84–1.07)	0.384
Dental appliance	137 (58.5)	36 (26.3)	101 (73.7)	1.00 (0.91–1.09)	0.956
Dysphagia risk					
EAT-10 score (mean ± SD)^c^	3.0 ± 5.8	1.4 ± 2.2	3.6 ± 6.5	0.98 (0.97–0.99)	< 0.0001**
BMI					
(mean ± SD)	24.7 ± 5.3	27.4 ± 5.2	23.7 ± 5.0	1.02 (1.02–1.03)	< 0.0001**
Muscle strength					
Hand grip, kg (mean ± SD)^d^	14.5 ± 7.0	16.0 ± 7.5	13.9 ± 6.7	1.01 (1.00–1.02)	0.006**
Requires help with ADL n (%)					
No	77 (32.9)	25 (32.5)	52 (67.5)	1	
Yes	157 (67.1)	37 (23.6)	120 (76.4)	0.93 (0.86–1.00)	0.061
Cognitive status					
MoCA score (mean ± SD)^e^	18.5 ± 5.8	20.3 ± 5.4	17.8 ± 5.8	1.01 (1.00–1.02)	0.025**
Number of medications					
(mean ± SD)	7.5 ± 4.4	7.1 ± 3.5	7.7 ± 4.6	1.00 (0.99–1.01)	0.537
Number of comorbidities					
(mean ± SD)	5.7 ± 2.7	5.6 ± 2.9	5.7 ± 2.7	1.00 (0.98–1.02)	0.683
Nutrition supplements n (%)					
Taking^f^	82 (35.0)	25 (30.5)	57 (69.5)	1	
Not taking	152 (65.0)	37 (24.3)	115 (75.7)	0.95 (0.87–1.02)	0.163

*MNA®-SF* Mini Nutritional Assessment Short-Form, *EAT-10* Eating Assessment Tool, *BMI* body mass index, *ADL* activities of daily living, *MoCA* Montreal Cognitive Assessment

^**a**^Descriptive statistics for continuous data were presented as mean ± standard deviation (SD) and categorical data presented as frequencies (n) and percentages (%)

^b^Poisson regression with robust variance estimation. Each parameter was adjusted for age, gender, ethnicity, number of comorbidities and medications; PR (95% CI) ** *p* value significant at *p* < 0.05

^c^Missing data n = 2

^d^Missing data *n* = 66

^e^Missing data *n* = 69

^f^Average number of supplements taken n = 1

After adjusting for age, gender and ethnicity, analysis from Poisson regression with robust variance estimation identified [PR (95% CI)] high dysphagia risk [EAT-10 score: 0.98 (0.97–0.99)], low muscle strength [hand grip strength (kg): 1.01 (1.00–1.02)], low BMI (kg/m^2^) [1.02 (1.02–1.03)] and decline in cognition [MoCA score: 1.01 (1.00–1.02)] as significant predictors of malnutrition risk in older adults at hospital admission (Table 2).

## Discussion

This is the first study in New Zealand to assess prevalence and predictors of malnutrition risk in older adults at hospital admission. Almost three-quarters were either malnourished (26.9%) or at malnutrition risk (46.6%), congruent with previously published international findings (15–78%) [13–21]. Dysphagia risk, low BMI, decreased muscle strength and cognitive decline, were identified as statistically significant predictors of malnutrition risk, at hospital admission. As 88% of participants were admitted from the community, this suggests that the high prevalence of hospital malnutrition may be a result of unrecognised community malnutrition; hence routine screening is essential on hospital admission. Furthermore, it suggests that community based assessment and intervention may be important for identifying at-risk adults and providing early supportive measures.

The prevalence of malnutrition (26.9%) observed in this study is congruent with previously reported hospital malnutrition prevalence (20–60%) internationally [7–9]. This suggests that both malnutrition risk and malnutrition are present in older adults prior to hospital admission. This study is an extension of our pilot investigation that reported malnutrition risk prevalence in adults (*n* = 88) of advanced age (85+ years), wherein a similar prevalence of malnutrition risk was observed [46]. Previously, malnutrition has been identified in New Zealand older adults (*n* = 55) hospitalised for hip fracture; where 42% were found to have at least two indices of protein energy malnutrition as evidenced by low triceps skinfold thickness, reduced mid-upper arm circumference, and low serum pre-albumin [47]. Moreover, using the MNA®-SF, a New Zealand hospital audit of older adults (*n* = 72) found 24% were malnourished and 44% were at nutrition risk [48]. The insights from the aforementioned reports and findings from our study suggest the need for routine screening at hospital admission in New Zealand.

Previous studies reported chewing and swallowing problems as common phenomena in older people, which may lead to decline in food intake [49–51]. Reduced food intake is one of the main causes of malnutrition. Thus, understanding both dental status and dysphagia risk may be helpful in devising strategies intended to prevent malnutrition. In contrast to previous studies [52, 53], no significant association between dental status and malnutrition risk status was observed in the current study. However, as expected, we observed that with increasing risk of dysphagia (increasing EAT-10 scores), the prevalence ratio of malnutrition risk also increased. This concurs with findings of a previous study that assessed the association between swallowing difficulties and hospital malnutrition [28]. In the current study, 22% of the participants demonstrated EAT-10 scores ≥3 which suggests that in older people who are malnourished or at malnutrition risk, consideration of swallow assessment would be prudent. This may identify contributory problems and define treatment targets. Furthermore, for treatment of malnutrition, it may be helpful if future studies investigate the clinically relevant cut-off points for the EAT-10 associated with malnutrition risk, since an EAT-10 score ≥ 3 is merely suggestive of dysphagia [39].

Greater muscle strength as indicated by greater hand grip strength was associated with lower malnutrition risk. Similar findings were observed in an Australian study that explored the potential of hand grip strength to independently predict nutrition status in a hospital population [54]. A Canadian study that assessed malnutrition at hospital admission observed hand grip strength to be an independent factor associated with hospital stay [14]. Several factors explain the occurrence of poor muscle strength in older adults and how they may create a vicious cycle resulting in malnutrition. Reduced physical activity and functional decline commonly observed in older adults contribute to muscle atrophy and poor muscle strength [55–57]. In addition, malnutrition promotes body weight loss (low BMI), particularly muscle mass loss, including the deglutitive muscles, which consequently increases swallowing problems and hence malnutrition risk [58, 59].

Muscle weakness and low BMI often occur together in undernourished people [60, 61]. Our study supports this association, as a positive correlation (*r* = 0.236, *p* < 0.046) between BMI and muscle strength was observed. BMI is an integral part of the MNA®-SF and in older adults high BMI values are associated with low malnutrition risk [28, 62], better functional status [63] and may be protective against mortality [62, 64, 65]. Accordingly, a BMI ≥23kg/m^2^ has been proposed as the healthy cut-off point for older adults [45]. In the current study, 41% of the participants had a BMI < 23 kg/m^2^ which may explain the high prevalence of malnutrition risk observed.

Two-thirds of participants required daily help with various tasks such as cooking, cleaning, showering and dressing. This may suggest loss of physical function among these participants which may contribute to the low muscle strength and high malnutrition risk observed. Previous studies reported that malnutrition risk is higher in older adults who rely on others for assistance with daily activities like grocery shopping and cooking [8, 14, 26]. Although not statistically significant (*p* = 0.061), our data demonstrated an increased risk of malnutrition among older adults who required help with ADL. Whether this incremental decrease in nutrition status can be detected clinically is not clear from this study.

Medications and comorbidities are well documented factors associated with malnutrition [8, 26]. In the current study, on average each participant had six comorbidities and was taking seven medications. Although these factors were not significantly associated with malnutrition risk in this study, they may contribute to decreased physical activity, decline in muscle mass and consequently poor muscle strength. These factors negatively influence malnutrition risk [58, 59]. Two-thirds (65%) of the participants did not take any nutritional supplements and among those who took supplements, on average only one supplement was taken. This may explain why taking nutrition supplements did not impact malnutrition risk. A recent study in New Zealand observed low micronutrient intake in older adults and noted that with some of the micronutrients, those who took nutritional supplements were less likely to be nutrient deficient [66]. Further education in this area may assist older adults in determining whether supplementation is appropriate.

In agreement with previous studies [26], malnutrition risk was associated with decline in cognitive status. Cognitive impairment is associated loss of independence which may negatively affect older adults’ lifestyle, specifically food intake and physical activity. Malnutrition and cognitive impairment have been reported as predictors of all-cause mortality among hospitalised adults (> 70 years) [67]. In the current study, 62% of the participants indicated some level of cognitive impairment (MoCA score ≤ 26). Although about a quarter of the participants declined taking the MoCA test, the high prevalence of cognitive impairment among the test takers suggests the importance of performing cognitive assessments in older adults.

The strengths of this study include the use of validated screening tool MNA®-SF and the use of a critical time point for data collection i.e. at hospital admission. These factors enable understanding of malnutrition risk in older New Zealanders at hospital admission, which provides helpful insights towards promoting good nutritional status not only during hospitalisation but also prior in the community and after discharge. However, the use of an observational study design hinders us from determining causality. Additionally, investigations were done using a convenience sample (*n* = 234), which limits generalisability of the findings. Hand grip strength and cognitive status assessments were undertaken with about three-quarters of the participants, therefore interpretation of these results should be made with caution, as the missing data may have introduced bias in our findings. Overall, the participants had low MoCA scores which may have influenced the malnutrition risk outcome, since some of the MNA®SF items rely on memory. Due to poor physical strength and/or presence of metals in the body, only 16 participants completed muscle mass measurement using bio-impedance analysis scale, thus we could not report muscle mass findings. As significant changes in body composition occur with ageing, inability to assess muscle mass in this study limited our ability to understand the impact of body composition on malnutrition risk. To understand participants’ general health status, the current study recorded comorbidities and medications taken. However, the study did not capture reason for admission, which would have helped clarify participants’ health upon admission.

## Conclusion

Among older adults recently admitted to hospital rehabilitation wards, almost three-quarters were malnourished or at malnutrition risk. This illustrates the need for timely screening, as early identification is one of the most important and effective ways to prevent and reduce the prevalence of malnutrition and malnutrition risk. As 88% of the participants were admitted from the community, this illustrates the need for routine nutrition screening both at hospital admission and in community-dwelling older adults. Routine screening, especially at hospital admission, would identify those that may benefit from intervention or nutritional support during hospitalisation. Interventions that focus on increasing muscle strength and/or preventing dysphagia risk, while considering the cognitive functioning of older adults may reduce malnutrition risk. This cross-sectional study indicates the need for robust intervention studies to investigate the efficacy of prevention or reversal of malnutrition risk factors on nutritional status.

## Abbreviations

ADL: Activities of daily living
BMI: Body mass index
CI: Confidence interval
EAT-10: Eating Assessment Tool
MNA®-SF: Mini Nutritional Assessment Short-Form
MoCA: Montreal Cognitive Assessment
PR: Prevalence ratio
SD: Standard deviation
